# Specificity of time- and dose-dependent morphological endpoints in the fish embryo acute toxicity (FET) test for substances with diverse modes of action: the search for a “fingerprint”

**DOI:** 10.1007/s11356-021-16354-4

**Published:** 2021-10-13

**Authors:** Rebecca von Hellfeld, Pauline Pannetier, Thomas Braunbeck

**Affiliations:** 1 Center for Organismal Studies, Aquatic Ecology and Toxicology Section, University of Heidelberg, Im Neuenheimer Feld 504, 69120 Heidelberg, Germany; 2 Present Address: University of Aberdeen, Institute of Biological and Environmental Science, 23 St Machar Drive, AB24 3UU, Aberdeen, UK

**Keywords:** Fish embryo toxicity test, Zebrafish, *Danio rerio*, OECD TG 236, Acute toxicity, Sublethal toxicity, Specificity, Sensitivity

## Abstract

The fish embryo acute toxicity (FET) test with the zebrafish (*Danio rerio*) embryo according to OECD TG 236 was originally developed as an alternative test method for acute fish toxicity testing according to, e.g., OECD TG 203. Given the versatility of the protocol, however, the FET test has found application beyond acute toxicity testing as a common tool in environmental hazard and risk assessment. Whereas the standard OECD guideline is restricted to four core endpoints (coagulation as well as lack of somite formation, heartbeat, and tail detachment) for simple, rapid assessment of acute toxicity, further endpoints can easily be integrated into the FET test protocol. This has led to the hypothesis that an extended FET test might allow for the identification of different classes of toxicants via a “fingerprint” of morphological observations. To test this hypothesis, the present study investigated a set of 18 compounds with highly diverse modes of action with respect to acute and sublethal endpoints. Especially at higher concentrations, most observations proved toxicant-unspecific. With decreasing concentrations, however, observations declined in number, but gained in specificity. Specific observations may at best be made at test concentrations ≤ EC_10_. The existence of a “fingerprint” based on morphological observations in the FET is, therefore, highly unlikely in the range of acute toxicity, but cannot be excluded for experiments at sublethal concentrations.

**Supplementary Information:**

The online version contains supplementary material available at 10.1007/s11356-021-16354-4.

## Introduction

In 2019, the European Union produced 277.8 million tons of hazardous chemicals (Eurostat 2020), and, according to CEFIC (2021) and Statista (https://www.statista.com/), the 2018 global chemical revenue amounted to approximately US $ 4100 billion. Together with a multitude of metabolites, most anthropogenic substances finally end up in the environment through unintended or incorrect use, uncontrolled disposal, incomplete elimination during wastewater treatment, and surface run-off (Andreozzi et al. 2003; Schock et al. 2012), thus leading to an unmanageable variety of contaminants in surface, ground, and drinking waters (Küster and Adler 2014). As a consequence, more recent legislation such as Registration, Evaluation, Authorization and Restriction of Chemicals (REACH; EU 2007) and novel testing programs such as the U.S. Environmental Protection Agency (EPA) ToxCast (Dix et al. 2007; Sipes et al. 2011; Padilla et al. 2012; Volz et al. 2015) prompted a massive increase of toxicity testing (EC 2020) and culminated in the quest for high-throughput assays (Rovida 2009, 2015; Hartung and Rovida 2009).

Since tests with vertebrates are an integral part of environmental hazard identification and risk assessment of chemicals, plant protection products, pharmaceuticals, biocides, feed additives, and effluents (Scholz et al. 2013), this increase in testing requirements has raised increasing concern about animal welfare (Braunbeck et al. 2005, 2015; Paparella et al. 2020; von Hellfeld et al. 2020). In order to meet these concerns, in 2003, Germany replaced whole effluent acute fish toxicity (AFT) testing according to OECD TG 203 (OECD 1992, 2019) with the zebrafish (*Danio rerio*) fish egg test (Bundesgesetzblatt 2005; ISO 2007), and, in 2013, the OECD adopted the fish embryo acute toxicity (FET) test (TG 236; (OECD 2013)) as an alternative method for the AFT test. According to current EU Animal Welfare Regulation (EU 2010), zebrafish embryos are not regarded protected according to current EU animal welfare legislation (Strähle et al. 2012).

In order to provide equivalent sensitivity to the AFT test (OECD 1992, 2019), the original FET test protocol (OECD 2013) was designed to use only 4 morphological core endpoints: coagulation of the embryo, lack of somite formation, lack of heartbeat, and non-detachment of the tail (OECD 2013). These endpoints were selected for (1) their direct or indirect association with mortality, (2) their practicality for screening by well-trained technical staff, and (3) their ease for recording and reporting. Over the last two decades, however, the zebrafish embryo has also been developed further into one of the most promising models not only in ecotoxicity testing (Braunbeck et al. 2015), but also in mammalian toxicology (Nagel 2002; Ton et al. 2006; Braunbeck 2009; Brannen et al. 2010; Sipes et al. 2011; Sukardi et al. 2011; Ali et al. 2011; De Esch et al. 2012; Driessen et al. 2013; Scholz et al. 2013; Nishimura et al. 2015; Guo et al. 2015; Bambino and Chu 2017; Fernandes et al. 2018). The versatility of the FET test has thus prompted a massive expansion of the scope of the FET test, which, in turn, led to the integration of numerous further endpoints into the original FET protocol and resulted in a rapidly growing list of not only morphological observations, but also physiological, biochemical, and molecular endpoints.

In fact, exposure of aquatic biota to environmental pollutants can lead to a multitude of specific or unspecific adverse effects, which may easily become relevant for the performance of populations via, e.g., feminization due to exposure to estrogenic compounds (Matthiessen et al. 2018; Wolf and Wheeler 2018; Dang and Kienzler 2019), or via behavioral changes due to neurotoxicity by heavy metals, organochlorine compounds, or pesticides (De Esch et al. 2012; Dhillon et al. 2015; Yueh and Tukey 2016; Nishimura et al. 2016; Green and Planchart 2018)). Whereas estrogen-receptor-mediated feminization is—by definition—a specific process, behavioral changes are likely to be unspecific (Tilton et al. 2011), unless target-specific molecular interactions like inhibition of enzymes such as acetyl choline esterase inhibition by phosphate ester pesticides and carbamates are concerned (Fulton and Key 2001; Behra 2004; Yen et al. 2011; Russom et al. 2014; Kais et al. 2015). The distinction between specific and unspecific endpoints may deepen the current understanding of adverse effects on populations and in risk assessment.

While most apical endpoints of acute toxicity are per se non-specific, tests addressing more specific endpoints such as endocrine disruption, genotoxicity, neurotoxicity, or immunotoxicity hold greater potential to yield specific reactions (Nendza and Wenzel 2006; Singh et al. 2019; Li et al. 2019). Especially with the advent of molecular techniques in (eco-)toxicology, the hypothesis developed that specific changes of a combination thereof might serve as a “fingerprint” of the contaminant or contaminant class (Peterson and Bain 2004; Yang et al. 2009; Gagné et al. 2013; Zhang et al. 2015; Neale et al. 2017). The massive diversification of FET test protocols has thus also led to the hypothesis that an extended FET test might allow for the identification of different classes of toxicants via a “fingerprint” of morphologically detectable observations. To test this hypothesis, the present study investigated 18 compounds with highly diverse modes of action with respect to acute and sublethal morphological endpoints in the FET test. In order to characterize the specificity of the morphological observations, data were analyzed not only with respect to their assignment to specific substances or substance classes, but also with regard to their time- and dose-dependence.

## Materials and methods

### Chemicals and test substances

Test compounds were selected for the diversity of their modes of action. Primary mode(s) of action as well as detailed information on the preparation of test solutions are summarized in Table 1. All compounds tested were purchased at a minimum purity of 98%. Paraquat, carbaryl, colchicine, rifampicin, clofibrate, sulfisoxazole, and taxol were obtained from Carbosynth (Compton, UK); rotenone, tebuconazole, and ibuprofen were obtained from TCI (Eschborn, Germany); and acrylamide, hexachlorophene, 1-methyl-4-phenyl-pyridinium iodide (MPP^+^), paracetamol, PCB 180, tolbutamide, triphenylphosphate, and valproic acid were purchased from Sigma-Aldrich (Deisenhofen, Germany). Dimethyl sulfoxide (DMSO) was ordered from Honeywell International (Offenbach, Germany). All test solutions were freshly prepared immediately prior to use in standardized water (ISO 1996); in cases of limited water solubility, DMSO was used as a solvent: clofibrate, hexachlorophene, rotenone, tebuconazole, tolbutamide, and valproic acid were dissolved in 0.1% DMSO, whereas carbaryl and ibuprofen were dissolved in 0.5% DMSO, which has been determined as an acceptable concentration for FET test experiments in previous studies (Maes et al. 2012; Christou et al. 2020). PCB 180, rifampicin, sulfisoxazole, and taxol were dissolved in 1% DMSO, since no adverse effects were observed at the highest test concentrations, when dissolved in 0.5% DMSO. When the DMSO concentration was even further increased to 1% and no effect was seen at the highest test concentration, these compounds were not tested further to avoid interference of DMSO toxicity with the observations (Table 1). Additional information on log *K*_OW_, solubility, and stability as well as application profiles and biological effects is provided in Supplemental materials Tables 1 and 2.

**Table 1:** Test compounds used in the acute fish embryo toxicity tests with the zebrafish (*Danio rerio*) embryo: media and exposure concentrations as well as primary mode(s) of action according to literature data

**Compound**	**Solution medium**	**Test concentrations (mg/L)**	**Mode(s) of action**	**References**
Acrylamide	H_2_O	43.75, 87.5, 175, 350	ED, MCI	Tyl and Friedman (2003), Faria et al. (2018)
Carbaryl	0.5% DMSO	1.89, 3.75, 7.5, 15	AI, CYP	Slaninova et al. (2009), Schock et al. (2012)
Clofibrate	0.1% DMSO	62.5, 125, 250, 500, 1000	PM, OX	Laville et al. (2004), Den Broeder et al. (2015)
Colchicine	H_2_O	10, 20, 40, 80	MT	Jesús et al. (1987)
Hexachlorophene	0.1% DMSO	2, 4, 8, 16, 32	MCI	Zheng et al. (2012)
Ibuprofen	0.5% DMSO	2.50, 5, 10, 20, 40, 80	PM, CI	David and Pancharatna (2009a), Puhl et al. (2015)
MPP^+^	0.1% DMSO	100, 200, 400, 800, 1600	HDAC	Pinho et al. (2016)
Paracetamol	H_2_O	125, 250, 500, 1000, 2000	OX, CI	David and Pancharatna (2009b), Du et al. (2016)
Paraquat	H_2_O	125, 250, 500, 1000	OX	Slaninova et al. (2009), Lushchak (2016)
PCB 180	1.0% DMSO	3.13, 6.25, 12.50, 25	cGMP	Llansola et al. (2009)
Rifampicin	1.0% DMSO	50, 100, 200, 400, 800	CYP	Mahatthanatrakul et al. (2007)
Rotenone	0.1% DMSO	2, 4, 8, 16, 32	MCI, OX	Cheng and Farrell (2007), Slaninova et al. (2009), Pinho et al. (2013), Wang et al. (2017)
Sulfisoxazole	1.0% DMSO	50, 175, 250, 500, 1000	BAC, CYP	Hong et al. (1995)
Taxol	1.0 % DMSO	6.25, 12.50, 25, 50	OX, MT	Brito et al. (2008), Lisse et al. (2016)
Tebuconazole	0.1% DMSO	1.88, 3.75, 7.50, 15, 30	ED, CYP, OX	Sancho et al. (2010), Yang et al. (2018)
Tolbutamide*	0.1% DMSO	57.34, 71.60, 89.60, 112, 140	MCI	Zhou et al. (2009)
Triphenylphosphate	0.1% DMSO	0.19, 0.38, 0.75, 1.50, 3	ED, CYP, PM	Isales et al. (2015), Du et al. (2016), Liu et al. (2016)
Valproic acid	0.1% DMSO	3, 7, 14, 29, 58, 115	HDAC, MCI	Chateauvieux et al. (2010), Godhe-Puranik et al. (2013)

Abbreviations: *AI* acetylcholine esterase inhibition, *BAC* bacterial inhibition, *cGMP* glutamate-NO-cGMP pathway inhibition, *CI* COX inhibition, *CYP* CYP450 inhibition, *ED* endocrine disruption, *HDAC* HDAC inhibition, *MCI* membrane channel inhibition, *MT* microtubule binding, *OX* oxidative stress, *PM* PPAR modulation

*pH of the final solutions had to be adjusted

### Fish maintenance

Adult wild-type zebrafish of the Westaquarium strain were obtained from breeding facilities at the Aquatic Ecology and Toxicology Group within the Centre for Organismal Studies (University of Heidelberg; licensed under no. 35-9185.64/BH). Fish maintenance, breeding conditions, and egg production were described in detail by Lammer et al. (2009) and are in accordance with internationally accepted standards.

### Fish embryo acute toxicity test (OECD TG 236)

The acute toxicity of the test substances was determined according to OECD TG 236 (OECD 2013). In brief, freshly spawned eggs (< 1 h post-fertilization (hpf)) were transferred to 50-ml crystallizing dishes filled with the respective test solutions. After control of the fertilization success, eggs were individually transferred to 24-well plates (TPP, Trasadingen, Switzerland) filled with 2 ml of test solution per well (1 embryo per well). All test vessels had been pre-incubated (saturated) with the test solutions for at least 24 h. Subsequently, well plates were sealed with self-adhesive foil (SealPlate® by EXCEL Scientific, Dunn, Asbach, Germany) and were placed in a Binder KT incubator (Tuttlingen, Germany) at 26.0 ± 1.0 °C under a 10/14-h dark/light regime. The test medium was renewed each day (semi-static exposure), and all developmental alterations of the embryos were documented at 24, 48, 72, and 96 hpf, according to OECD TG 236 (OECD 2013) and Nagel (2002), respectively. FET tests with a minimum mortality rate of 30% in the positive control (4 mg/L 3.4-dichloroaniline (DCA)) and a maximum effect rate of 10% in the negative control (dilution water) at 96 hpf were classified as valid.

In addition to the endpoints specified by OECD TG 236, namely (1) coagulation of fertilized eggs, (2) lack of somite formation, (3) non-detachment of tail bud, and (4) lack of heartbeat (OECD 2013), any other observation was recorded as further lethal or sublethal morphological endpoints: Common examples were reduced heartbeat or reduced blood flow, inhibited or missing pigmentation, delayed or altered development, modified movement(s), distortion of the spine, craniofacial deformations, eye development, delayed hatching and fin formation, and formation of various types of edema (von Hellfeld et al. 2020). In the case of evidence for delayed toxicity, the standard exposure duration of 96 h specified by OECD TG 236 (OECD 2013) was extended to 120 h. In any case, the developmental stage at the end of the experiments never exceeded the limits for unprotected developmental stages set by the current EU animal welfare legislation (EU 2010; Strähle et al. 2012). In case 2 range-finding experiments already provided conclusive results, only two replicates of the definitive FET test with 5 test concentrations spaced by a factor not larger than 2 were conducted for each compound. Otherwise, 4 replicates of the full FET test were conducted. The embryos were analyzed individually under an Olympus CKX41 inverted microscope (Olympus, Hamburg, Germany), and images were captured using an Olympus C5040 AUD camera.

### Data analysis and statistics

Lethal concentrations (LC) for the 4 core endpoints listed in OECD TG 236 (OECD 2013) as well sublethal effect concentrations (EC) based on the 4 core endpoints *plus* any other effect were calculated at levels of 10 and 50% based on probit analysis using linear maximum likelihood regression with ToxRat® (ver. 2.10.06; ToxRat^TM^ Solutions, Alsdorf, Germany), with both lethal and sublethal effects included into the calculation of EC values (Hrovat et al. 2009). The relative frequencies of morphological observations were calculated as follows: The percentage of coagulated embryos was calculated based on the total number of individuals of the given concentration, whereas the percentage of the other 3 lethal endpoints (lack of somite formation, tail detachment, and heartbeat) was computed based on the number of non-coagulated individuals. Relative percentages for sublethal effects were calculated on the basis of surviving embryos. Data were also analyzed for time-dependent changes in LC values for 72-h-old embryos via ANOVA-on-ranks (Kruskal-Wallis) followed by Dunn’s post hoc test, as included in SigmaPlot Version 13.0.0.83 (Systat-Jandel, Erkrath, Germany).

## Results and discussion

### Formal lethal and sublethal toxicity data from the standard fish embryo acute toxicity test (OECD TG 236)

Out of the 18 compounds tested, 13 expressed morphologically observable effects in the FET test (Table 2). Although tested with a maximum final DMSO concentration of 1%, PCB 180, rifampicin, sulfisoxazole, and taxol did not produce any effect up to the highest concentrations tested (cf. Table 1). Likewise, MPP^+^ was tested to a final concentration of 1.6 g/L, but failed to induce any morphological sign of toxicity and was, therefore, excluded from further analysis.

**Table 2:** Subletal and acute toxicity of selected test compounds (EC and LC values at 10 and 50% effect levels) in embryos of the zebrafish (*Danio rerio*) at 96 hpf

	**EC**_**10**_	**EC**_**50**_	**LC**_**10**_	**LC**_**50**_
Acrylamide (mg/L)	75.5 ± 13.09	94.3 ± 7.8	154.6 ± 53.2	205.7 ± 3.1
Carbaryl (mg/L)	2.2 ± 0.3	2.4 ± 0.2	6.6 ± 1.6	12.2 ± 0.7
Clofibrate (mg/L)	213.3 ± 36.7	342.5 ± 53.9	602.7 ± 36.1	1113.2 ± 23.6
Colchicine (mg/L)	23.1 ± 3.9	32.4 ± 2.9	32.5 ± 8.4	41.4 ± 6.5
Hexachlorophene (μg/L)	4.0 ± 0.1	5.0 ± 0.1	7.0 ± 0.3	8.0 ± 0.3
Ibuprofen (mg/L)	4.7 ± 1.5	10.8 ± 2.9	31.7 ± 2.1	37.3 ± 3.5
Paracetamol (mg/L)	219.8 ± 2.9	262.6 ± 2.4	1000 ± 0.1	1167.5 ± 3.1
Paraquat (mg/L)*	384.7 ± 64.3	545.9 ± 7.2	721.1 ± 8.1	855.0 ± 4.8
Rotenone (μg/L)	4.0 ± 0.1	7.1 ± 1.0	6.0 ± 1.2	10.0 ± 2.3
Tebuconazole (mg/L)	2.3 ± 0.1	5.3 ± 0.2	15.0 ± 0.1	17.3 ± 0.1
Tolbutamide (mg/L)	54.3 ± 12.3	116.9 ± 14.1	223.2 ± 6.9	278.6 ± 8.4
Triphenylphosphate (mg/L)	0.3 ± 0.1	0.5 ± 0.1	1.4 ± 0.1	1.6 ± 0.1
Valproic acid (mg/L)*	5.0 ± 0.8	7.8 ± 1.0	33.7 ± 7.8	37.4 ± 2.9

*Exposure duration extended to 120 hpf

The two most toxic compounds were rotenone and hexachlorophene, both with EC_10_ values of 4 μg/L (± 0.3 and ± 0.1 μg/L, respectively). Apart from paraquat, the remaining pesticides also produced sublethal effects at very low concentrations, whereas the pharmaceuticals caused toxic effects at highly variable concentration levels. The EC_10_ of valproic acid, e.g., was found to be 5.0 ± 0.7 mg/L, while EC_10_ values for paracetamol and clofibrate were > 200 mg/L (± 2.9 and ± 36.7 mg/L, respectively); trends for lethal toxicity (LC) values were similar. As a rule, toxicity data for pharmaceuticals also spanned a larger range (flat slope of the concentration-response relationship): For instance, ibuprofen had an EC_10_ value of 4.7 ± 1.47 mg/L and an LC_50_ value of 37.3 ± 3.48 mg/L, and valproic acid ranged from 5.0 ± 0.73 mg/L (EC_10_) to 37.4 ± 2.91 mg/L (LC_50_). In contrast, the concentration-response relationship of the insecticide carbaryl displayed a much steeper slope, i.e., sublethal and lethal toxicity data were much closer (EC_10_, 2.2 ± 0.26 mg/L, and LC_50_, 12.2 ± 0.72 mg/L). For further details and analyses of LC and EC data in zebrafish embryos, see von Hellfeld et al. (2020), where FET observations were discussed in the context of a catalog of FET endpoints.

### Further FET test observations and their potential for substance specificity

Morphological observations recorded throughout the 96 h of exposure were categorized into (1) a group of clearly “sublethal effects” (occurring at concentrations < EC_50_, Table 3) and (2) a group of endpoints recorded at a concentration between EC_50_ and LC_50_ values (“lethal effects,” Table 4). Given that the 4 core endpoints listed by OECD TG 236 had been selected as a clear indicator of mortality, these could only rarely be recorded at exposure concentrations < EC_50_; in fact, only coagulation and missing heartbeat could be identified at concentrations (< 10% of individuals) below which no other endpoint was positive (Table 3); in such cases, acute lethality drives the lowest observed effect concentration (LOEC).

**Table 3:** Frequencies (% of individuals) of sublethal observations in zebrafish (*Danio rerio*) embryos at concentrations ≤ EC_50_ values at 96 hpf

		**OECD 236 endpoints**		**Unspecific endpoints**										
	**EC**_**50**_ **(mg/L)**	**Coagulation**	**Missing heartbeat**	**Delayed development**	**Impaired heartbeat**	**Impaired/missing blood flow**	**Pericardial edemata**	**Impaired pigmentation**	**Craniofacial deformation**	**Lordosis**	**Fin underdevelopment**	**Reduced yolk resorption**	**Tremor**	**Lack of hatching**
Acrylamide^#^	94.3	3	5	20	6	3	8		8	27				5/6
Carbaryl	2.4	3		3			3					3		
Clofibrate	0.24	3								5				
Colchicine^#^	32.4	7.5	3				3		3	3				
Hexachlorophene	0.01						3							
Ibuprofen	10.8	3		10					10	3/18			7.5	3/5
Paracetamol	260	3/10						20 /100	14/25		50	3		3/40
Paraquat^$^	546								5	5		5	10	
Rotenone	0.01				10		10							5
Tebuconazole	5.3			3					3					14/18
Tolbutamide	117								8					8/11
Triphenylphosphate	0.5			5					3					3/15
Valproic acid^$^	7.8	3	3			3/13	8						3	27

In cases where 2 test concentrations were below the EC_50_ value, frequencies for both test concentrations are given, starting with the higher concentration

^$^Exposure duration extended to 120 h

^#^For acrylamide and colchicine, only 10 individuals per replicate were tested

**Table 4:** Frequencies (% of individuals) of lethal observations in zebrafish (*Danio rerio*) embryos at concentrations between EC_50_ and LC_50_ values at 96 hpf

	**LC**_**50**_ **(mg/L)**	**OECD 236 endpoints**		**Unspecific endpoints**												**More specific endpoints**		
		**Coagulation**	**Missing heartbeat**	**Delayed development**	**Impaired heartbeat**	**Impaired/missing blood flow**	**Pericardial edemata**	**Yolk edemata**	**Impaired pigmentation**	**Craniofacial deformation**	**Fin underdevelopment**	**Lordosis**	**Reduced yolk resorption**	**Tremor**	**Lack of hatching**	**Increased eye size**	**Scoliosis**	**Impaired tailfin**
Acrylamide^#^	205.7		50	38			28					20			13			
Carbaryl	12.2	3/15		8/31	46	41	5	24	3	3		33/65	45/65	45/61	5			
Clofibrate	1.1	3	3/15	3.5	3/50	3/50	5/43	3		5/63	3	11/21		4/22				
Colchicine^#^	41.4	8	35		17.5	17	6					21						23
Hexachlorophene	0.01	3	18.5	63	32	79	66				8.5	25			75			
Ibuprofen	37.3			8	3	35	33			5		5		43	68			
Paracetamol	1170		10		95	100	25		94.5	64	50	53			56	100		
Paraquat^$^	855									5		5	5	10				
Rotenone	0.01	25	10		17		39								12			
Tebuconazole	17.3		10		3/92	86	47			18		3		23	53/89			
Tolbutamide	278.6	3								41 /1		5.5	25		6			
Triphenylphosphate	1.6	5	63	5	97	94	67					3/7	32		43/90			
Valproic acid^$^	37.4	5		50	20/28	67/88		23/8	3	18/93	3	5/48	97/100	52/80	3		20	50

In cases where 2 test concentrations were below the EC_50_ value, the frequencies for both test concentrations are given, starting with the lower concentration

^$^Exposure duration extended to 120 h

^#^For acrylamide and colchicine, only 10 individuals per replicate were tested

In the present study, by definition, observations recorded after exposure to more than 4 of the separately tested substances (> 30% of the toxic substances) were classified as “unspecific,” if this observation was true at lethal concentrations. Here, all non-OECD endpoints observed at concentrations < EC_50_ were either induced by exposure to at least 4 of the tested compounds or represented different aspects of the same impaired system (e.g., missing heartbeat and reduced heartbeat). They were thus all classified as unspecific and occurred at low frequencies. As could be expected, the number of effects increased with the transition from effects < EC_50_ (Table 3) to effects between EC_50_ and LC_50_ (Table 4), i.e., with increasing concentration (positive concentration-response relationship). In parallel, the number of individuals affected (frequencies of occurrence) increased.

The most frequent observations recorded at sublethal concentrations (Table 3) were craniofacial deformation and lack of hatching (8 compounds), followed by coagulation (7 compounds) and formation of pericardial edemata (6 compounds). The endpoints observed with the lowest number of test compounds were impaired pigmentation and impaired fin development (1 compound each). Further endpoints observed less frequently were effects in the circulatory system such as impaired heartbeat and blood flow (2 compounds), as well as lack of heartbeat, reduced yolk resorption, and tremor (3 compounds). Overall, more adverse endpoints such as lack of blood flow, blood congestion, lordosis, and the OECD TG 236 core endpoints were less frequently observed.

As a rule, both numbers and frequencies of positively recorded endpoints decline with test concentrations. A remarkable exception is paracetamol (Table 3): At only ≤ EC_50_ concentrations, coagulation (in 3 and 10% of the exposure groups) and reduced yolk resorption (3%) could be observed. A high number of individuals also displayed impaired pigmentation (up to 100%, which declined to 94% at concentrations between EC_50_ and LC_10_). For most other endpoints, the frequencies of observations were limited to ≤ 20%, (exception: 27% lordosis with acrylamide). Overall, given the increasing lack of systemic responses at concentration levels well below EC_50_ values, the number of potentially more specific effects increases, an aspect that will be discussed further, when the time dependence of effects will be considered (Tables 5, 6, 7, and 8).

**Table 5:**
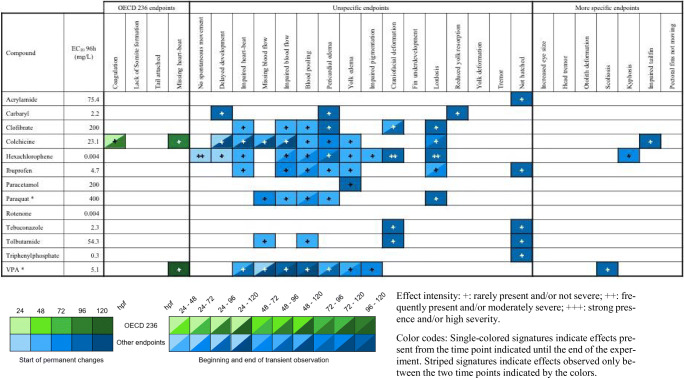
Semi-quantitative evaluation of all effects in zebrafish (*Danio rerio*) embryos observed at ≤ EC_10_ concentrations, grouped into lethal effects specified by OECD TG 236 (OECD 2013), unspecific alterations observed with most substances as well as “more specific” endpoints seen with < 4 substances. Compounds organized alphabetically (observations from *n* = 2 independent replicates)

Effect intensity: +, rarely present and/or not severe; ++, frequently present and/or moderately severe; +++, strong presence and/or high severity. Color codes: single-colored signatures indicate effects present from the time point indicated until the end of the experiment. Striped signatures indicate effects observed only between the two time points indicated by the colors

**Table 6:**
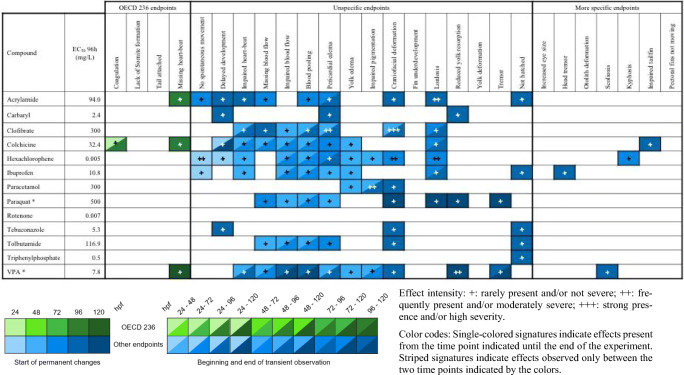
Semi-quantitative evaluation of all effects in zebrafish (*Danio rerio*) embryos observed at ≤ EC_50_ concentrations, grouped into lethal effects specified by OECD TG 236 (OECD 2013), unspecific alterations observed with most substances, and “more specific” endpoints seen with < 4 substances. Compounds organized alphabetically (observations from *n* = 2 independent replicates)

Effect intensity: +, rarely present and/or not severe; ++, frequently present and/or moderately severe; +++, strong presence and/or high severity. Color codes: single-colored signatures indicate effects present from the time point indicated until the end of the experiment. Striped signatures indicate effects observed only between the two time points indicated by the colors

**Table 7:**
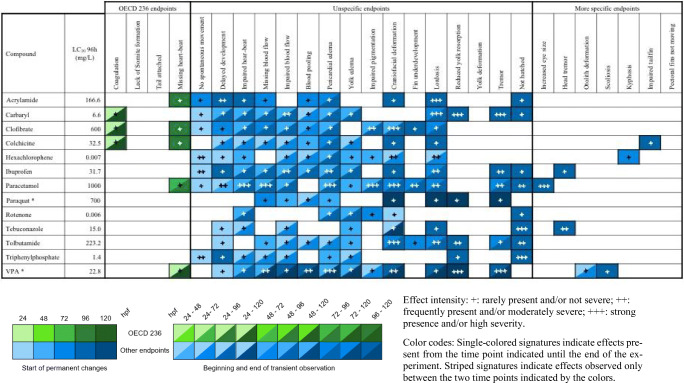
Semi-quantitative evaluation of all effects in zebrafish (*Danio rerio*) embryos observed at ≤ LC_10_ concentrations, grouped into lethal effects specified by OECD TG 236 (OECD 2013), unspecific alterations observed with most substances, and “more specific” endpoints seen with < 4 substances. Compounds organized alphabetically (observations from *n* = 2 independent replicates)

Effect intensity: +, rarely present and/or not severe; ++, frequently present and/or moderately severe; +++, strong presence and/or high severity. Color codes: single-colored signatures indicate effects present from the time point indicated until the end of the experiment. Striped signatures indicate effects observed only between the two time points indicated by the colors

**Table 8:**
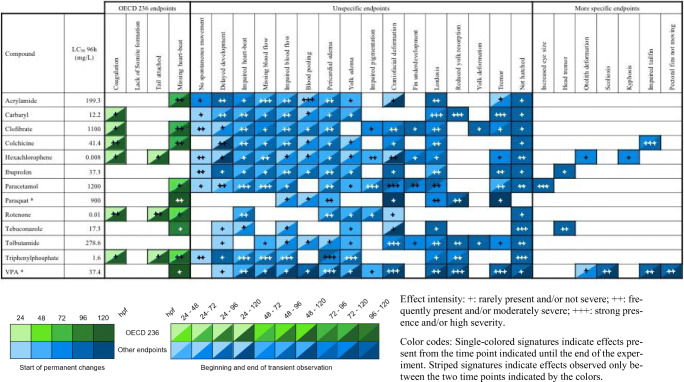
Semi-quantitative evaluation of all effects in zebrafish (*Danio rerio*) embryos observed at ≤ LC_50_ concentrations, grouped into lethal effects specified by OECD TG 236 (OECD 2013), unspecific alterations observed with most substances as well as “more specific” endpoints seen with < 4 substances. Compounds organized alphabetically (observations from n = 2 independent replicates)

Effect intensity: +, rarely present and/or not severe; ++, frequently present and/or moderately severe; +++, strong presence and/or high severity. Color codes: single-colored signatures indicate effects present from the time point indicated until the end of the experiment. Striped signatures indicate effects observed only between the two time points indicated by the colors

At concentrations between EC_50_ and LC_50_, some observations were encountered at higher frequencies (Table 4). The most commonly observed endpoint was lordosis (12 compounds), followed by impaired heartbeat, pericardial edemata, and lack of hatching (10 compounds). Further, common endpoints include impaired heartbeat (9 compounds) and coagulation, lack of heartbeat, and craniofacial deformation (8 compounds). In contrast, endpoints termed “more specific” in Table 4 were caused by a maximum of 2 compounds. Yolk edemata and impaired pigmentation were induced by 3 compounds, followed by impaired fin development (4 compounds).

Overall, frequencies of effects recorded between EC_50_ and LC_50_ are more diverse (3–100 %) than frequencies of observation ≤ EC_50_. As a rule, the number of effects between EC_50_ and LC_50_ is higher than at concentrations ≤ EC_50_. Among the core OECD TG 236 endpoints, lack of heartbeat was most frequent (acrylamide, 50%). For commonly observed non-OECD endpoints, few compounds exceeded 50% of individuals; again, paracetamol and valproic acid are exceptions (100 %) for impaired/missing blood flow and reduced yolk resorption, respectively. Relatively high prevalence of changes in the circulatory system and edema indicate that these effects lose specificity with increasing test concentrations.

### Time and severity dependence of observations in the FET

In contrast to a previous communication, which aimed at compiling a comprehensive and standardized catalog of observations in the FET test (von Hellfeld et al. 2020), the present study was designed to also analyze the onset and frequency (severity) of observations. Table 5 summarizes observations made at test concentrations ≤ EC_10_. The “more specific endpoints” found with only a low number of test compounds were considered as candidates for a fingerprint of toxicity, since these effects were indicative of the onset of compound-specific pathologies and were only rarely observed throughout the experiment. In fact, only 3 out of 7 “more specific” endpoints proved positive—all at very low frequencies and only showing up late (96–120 hpf). Likewise (and expectedly), OECD TG 236 core endpoints were also seen only rarely and at low frequencies. As a remarkable exception, colchicine exposure induced coagulation not only at the usual time point of 24 hpf, but also at later developmental stages (color code, 24–120 hpf).

The only endpoints induced at moderate frequency/severity were craniofacial deformation and lordosis after exposure to hexachlorophene. All other observations were made at low frequencies, with colchicine and hexachlorophene inducing the highest number of effects (11 each). With respect to the developmental phase, the majority of endpoints positive at ≤ EC_10_ was recorded at ≥ 48 hpf, and their partially transient nature was indicated by 80 % of the observations only being present for one specific time point.

Table 6 compiles all observations recorded at ≤ EC_50_ concentrations, including those listed in Table 5. With clofibrate-induced craniofacial deformation, the first observation with high severity/frequency (+++) is listed in Table 6. Out of the 84 observations recorded throughout this study, only 6 could be classified as moderately severe/frequent (++). Again, the majority of observations were made at ≥ 48 hpf, with only 30 listed for more than 1 time point.

Early developmental effects seen at ≤ EC_50_ concentrations were coagulation (colchicine), lack of spontaneous movement (hexachlorophene, ibuprofen), and delayed development (hexachlorophene). At concentrations between EC_10_ and EC_50_ values, the number of effects by acrylamide increased by 9. Valproic acid–exposed individuals were listed with 13 endpoints, as compared to 9 noted at ≤ EC_10_ (Table 5). Ibuprofen exposure induced another 3 effects (one of which was considered more specific), followed by paracetamol, paraquat, and tolbutamide (+ 2).

In contrast, only 3 out of the 6 endpoints listed for clofibrate ≤ EC_10_ were seen between EC_10_ and EC_50_, with a general delay of development as a new endpoint. For carbaryl, clofibrate, hexachlorophene, and triphenylphosphate, no changes were seen with respect to the type and number of endpoints from ≤ EC_10_ to ≤ EC_50_. Since for rotenone both EC_10_ (Table 5) and EC_50_ values (Table 6) were extrapolated, no observations are listed in either table.

Table 7 lists all observations recorded up to LC_10_ concentrations. If compared to Table 6 and, even more so, Table 5, the number of and time span for observations increase signficantly. In fact, except for lack of spontaneous movement, which is, almost by definition, restricted to 24 hpf, most endpoints proved positive (i.e., persistent) over extended periods of development. Thus, with increasing concentration, endpoints of diverse nature gradually accumulate, making these endpoints less specific of the test substance. On the other hand, the number of “more specific endpoints” (seen with < 4 substances) also increases. A conspicuous example of such “uncommon” observations is head tremor, which could only be seen after exposure to ibuprofen and tebuconazole. This endpoint has been speculated to be an indicator of reduced oxygen availability and has frequently been described as “gulping for air” (Huang et al. 2014). Usually, e.g., with paracetamol, but not necessarily, the severity of expression increases in parallel to test substance concentration and frequency of observations.

The trends seen in Table 7 find their continuation in Table 8: Most observations listed for test concentrations up to LC_50_ levels eventually lose all specificity. None of the test substances induced less than 8 endpoints, which was seen after expsoure to paraquat. In fact, hexachlorophene and valproic acid produced up to 18 different morphological effects in the extended FET test protocol; most interestingly, valproic acid also induced a total of 4 “more specific” endpoints, which was not expected at lethal concentrations close to LC_50_.

### Examples of substance-related effect profiles/specificity

The most effect-specific compounds from the present compound list are seemingly carbaryl and triphenylphosphate, as they induced the same few endpoints at EC_10_ and EC_50_. While neither of these endpoints, at sublethal concentrations, were considered specific, the fact that even ≤ EC_50_ for each of the compounds, no new endpoints became evident, this allows for the hypothesis that such endpoints are relatively compound-specific and should be assessed in light of the compound’s functioning and mechanisms of action.

#### Cabaryl

The endpoints which can be observed at sublethal concentrations were delayed development, pericardial edemata, and reduced yolk resorption. Previous research showed that carbaryl competitively binds to melatonin receptors (Popovska-Gorevski et al. 2017), negatively affecting the overall metabolism as well as the circadian clock. Reduced yolk resorption was hypothesized to be caused by alterations in lipid metabolism and PPARα expression levels (Weston et al. 2009; Coimbra et al. 2015; Kamstra et al. 2015). While in zebrafish embryos the liver is not fully formed (but active) before 72 hpf (de Esch et al. 2012), endpoints relating to the yolk sac are nonetheless correlated to the hepatic system and thus hold the potential to indicate hepatotoxic compounds. Thus, the observation of both reduced yolk resorption and delayed development can be deemed to be indicators of the compound’s effect on melatonin receptors. The development of pericardial edemata (with edemata being generally defined as a swelling of any body part due to fluid build-up; IQWiG 2016) is not fully understood. However, edemata observed in hexachlorophene-exposed mice proved to be only short-term effects and vanished after cessation of the treatment (Powell et al. 1973). It could thus be assumed that edemata, also observed to be induced by the exposure to 6 other compounds ≤ EC_10_, is an easily elicited response by the organisms which indicates an overall state of stress without being specifically linked to an underlying mechanism.

#### Triphenylphosphate

The only endpoint that could be recorded at ≤ EC_50_ for triphenylphosphate in the present study was the observation that embryos failed to hatch by 96 hpf (controls, 72 hpf; Kimmel et al. 1995), which might be linked to the following two general pathologies: (1) First, the inability to hatch might be based on physical developmental delay, which already becomes evident in, e.g., the lack or delay of tail detachment at 24 hpf (Kimmel et al. 1995). (2) Second, early spontaneous movement is thought to be an essential precoursor of hatching behavior (Xia et al. 2017). Embryonic behavioral endpoints such as coiling and swimming behavior have frequently been utilized to determine the developmental neurotoxic potential of compounds (Schmitt and Dowling 1999; Selderslaghs et al. 2010, 2013; Velki et al. 2017; Vliet et al. 2017; Ramlan et al. 2017; Basnet et al. 2019; Zindler et al. 2019b, a) and have successfully revealed alterations at sublethal concentrations. In the case of elevated (lethal) concentrations of triphenylphosphate (Tables 7 and 8), a multitude of unspecific endpoints including “tail non-detached,” “delayed development,” and “no spontaneous movement” could be listed and might be linked to either possible pathway of pathology. However, since the exposure failed to induce “tremor” as a further indicator of neurotoxicity (von Hellfeld et al. 2020), the observed lack of hatching at lower concentration was more likely due to developmental delays, which only become macroscopically visible at higher toxicant concentrations.

#### Acrylamide

Acrylamide-exposed zebrafish expressed an inability to hatch at ≤ EC_10_ as well as various circulatory defects (reduced heartbeat and impacted blood flow along with blood congestion) at sublethal concentrations, as well as delayed development and reduced hatching success. Previous studies found that acrylamide reduces the number of cardiomyocytes and their proliferative capacity, leading to morphological changes of the heart (Huang et al. 2018), thus impacting the general circulation and thus overall development. Whereas reduced heartbeat rates per se may not indicate direct pathology, considering it in correlation with endpoints such as reduced heart size or lack of heart looping, it may indicate a reduced proliferative capacity of the heart (Schock et al. 2012; Isales et al. 2015; Huang et al. 2018).

#### Colchicine

Exposure to the mitosis inhibitor colchicine produced pronounced lordosis in zebrafish embryos. Colchicine generally affects cell division, which may easily explain the incorrect cell formation associated with lordosis and finally coagulation of the embryo (even at time points later than 24 hpf) at ≤ EC_10_, as well as the impairment observed for tailfin development. In general, lordosis is also thought to be caused by alterations in the expression of the fibroblast growth factor, sonic hedgehog and bone morphogenetic protein, as well as *Wnt* and *Notch* genes (Lin 2002). The early onset of lordosis and coagulation at later developmental stages may thus seem specific of colchicine in the present study due to a combination of early pathways of developmental pathology.

#### Hexachlorophene

Even at ≤ EC_10_, hexachlorophene exposure induced kyphosis in the zebrafish embryos, along with various “unspecific” effects pertaining to the circulatory system, craniofacial formation, and lordosis. In contrast to lordosis as an inward concave curving of the cervical and lumbar regions of the spine, kyphosis is an abnormally excessive convex curvature of the spine especially in the thoracic and sacral regions. Kyphosis is thought to relate to myocyte degeneration and neural cell apoptosis (Kim et al. 2009). Hexachlorophene is a membrane channel inhibitor (Zheng et al. 2012), and it has been shown that the disruption of ion channel functionality plays a vital role in apoptosis (Kondratskyi et al. 2015), thus supporting the feasibility of kyphosis being more specific processes present in only certain cases. While the underlying pathways of spinal deformations such as lordosis, kyphosis, and scoliosis are yet to be fully elucidated, the differential observation of kyphosis, lordosis, and scoliosis (sideways curvature of the spine) offers an insight into potential pathways, which highlight the importance of correct identification and terminology of these endpoints due to their specificity (von Hellfeld et al. 2020).

#### Ibuprofen and tebuconazole

Both of these compounds induced the “head tremor” endpoints, which have previously been linked to a “gulping for air”-like behavior (Huang et al. 2014). Ibuprofen is a PPARα modulator and COX inhibitor (David and Pancharatna 2009a; Puhl et al. 2015) and induced the endpoint at ≤ EC_50_, whereas tebuconazole, an inducer of oxidative stress, endocrine disruptor, and CYP450 inhibitor (Sancho et al. 2010; Yang et al. 2018) only did so at LC_10_ concentrations. Oxidative stress has previously been identified as both a cause for and a consequence of reduced oxygen availability in fish, leading to the increased gill movement or “gulping” observed in the present study. Studies have shown that nonsteroidal anti-inflammatory drugs (NSAIDs) such as ibuprofen increase the cardiac output in fish (Zhang et al. 2020), thus leading to an increased need for oxygen to sustain this behavior. Thus, although “head tremor” endpoint is not a frequent observation, it may well have two distinct underlying mechanisms.

#### Paracetamol

The increased eye size following paracetamol exposure has been speculated to be caused by alterations in the retinoic acid pathway (Drummond and Davidson 2016), which is known to also induce heart deformations and damage to the retina (Isales et al. 2015). This led to the assumption that eye deformation, especially when observed along with heart deformation, indicates disruption of the retinoic pathway.

#### Valproic acid

Exposure to valproic acid induced a total of four “more specific” endpoints: otolith deformation, scoliosis, impaired tailfin development, and the lack of pectoral fin movement. Valproic acid is a known histone deacetylase (HDAC) inhibitor, which is required for the formation of the inner ear and other craniofacial structures (He et al. 2016), thus providing an explanation for the observation of otolith deformation. HDAC inhibition has further been linked to alterations in skeletal development in general and bone strength in mammals in particular. The inhibition of sirtuins (Bradley et al. 2015), a sub-group of HDAC enzymes, and HDAC2 (Tassano et al. 2015) in particular are known for inducing spinal curvature defects such as scoliosis. Further studies revealed that HDAC8 inhibition leads to smaller hands and feet in humans (Deardorff et al. 2012; Kaiser et al. 2014), while HDAC4 inhibition induced shortened metatarsals and metacarpals (Williams et al. 2010; Villavicencio-Lorini et al. 2013). While all these findings are based on humans and other terrestrial mammals, the genetic homology of zebrafish allows for the consideration that these underlying functions of the different HDACs are comparable to at least a certain degree, thus possibly explaining the unique tail and pectoral fin alterations observed in the present study.

### Time-dependent toxicity profile

**Fig. 1:**
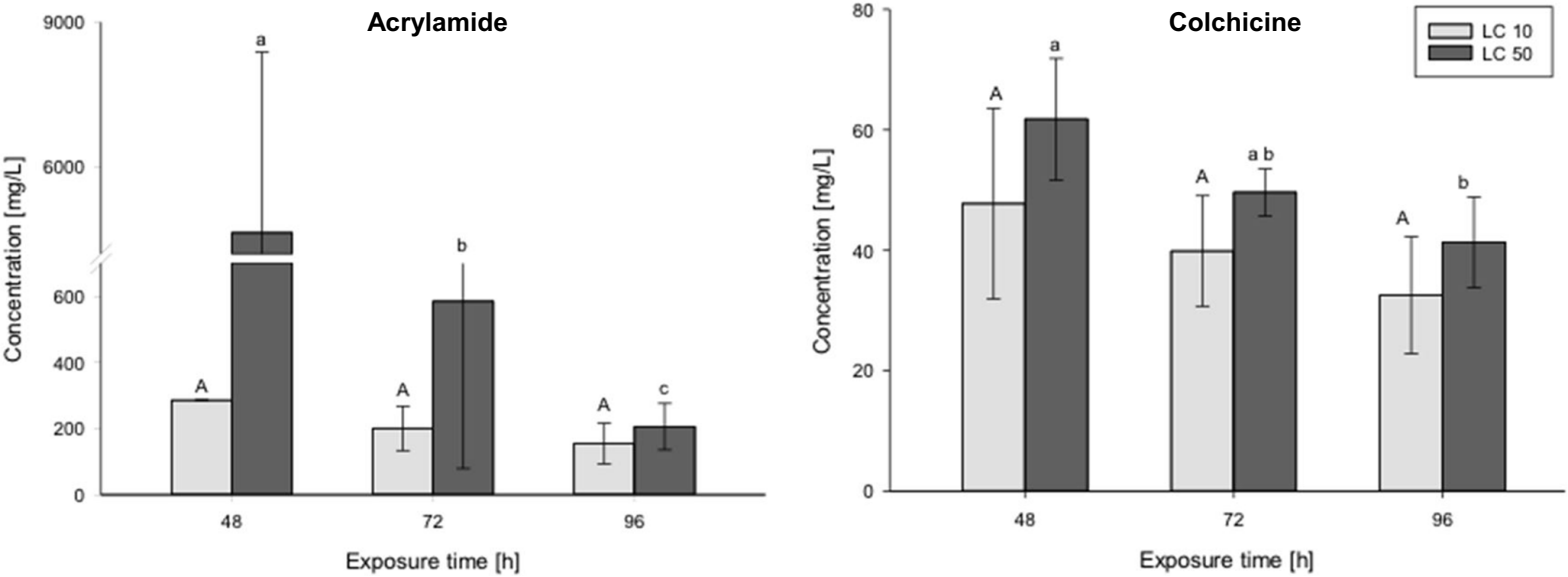
Time course of the toxicity of acrylamide (a) and colchicine (b) to embryos of zebrafish (*Danio rerio*) over 96 h according to OECD TG 236 (*n* = 4). Statistical significance is indicated by lowercase letters between time points (acrylamide: *ρ* = 0.034; colchicine: *ρ* = 0.019); identical capital letters indicate no statistically significant difference between time points

Out of the compounds tested in the present study, only acrylamide and colchicine expressed a statistically significant modulation of LC values over exposure time (Fig. 1). The LC_50_ of acrylamide significantly decreased between 48 and 96 hpf, with significant differences between each of the three time points tested (*ρ* = 0.034). In contrast, LC_10_ values for either substance did not show any significant impact over time, although there was a clear trend for colchicine: For individuals exposed to colchicine, the LC_50_ also varied significantly between 48 and 96 hpf (*ρ* = 0.019), yet without a significant difference between 48 and 72 hpf or 72 and 96 hpf.

The fish embryo test with the zebrafish embryo is conducted during a period of rapid development and, thus, massive time-dependent changes (Kimmel et al. 1995). Chemical compounds may affect mechanisms or organs, which have not fully developed: Hepatotoxic compounds, e.g., can only express their impact after 72 h of exposure, when the liver becomes functional. The full toxic potential on the zebrafish embryo, however, will likely unfold after 120 hpf, when the liver has reached full functionality and a certain volume (de Esch et al. 2012). This may, in part, provide an explanation why the toxicity of, e.g., valproic acid shows an increase over the entire exposure duration (Dai et al. 2015).

Another important consideration with respect to the time dependence of both morphological and functional effects is the potential barrier function of the chorion in combination with limited absorption rate and poor membrane permeability for certain compounds such as colchicine (Brox et al. 2016). As a consequence, a delay in the expression of toxicity may develop, which, however, can rapidly be compensated upon hatching (Roche et al. 1994; Henn and Braunbeck 2011). However, it should be noted that the barrier function of the chorion plays a less important role (Zhang and Rawsom 1996; Kais et al. 2013; Braunbeck et al. 2020) than originally postulated (Hagedorn et al. 1997, 1998; Adams et al. 2005). In line with the developmental time line, the nervous system is assumed to be fully developed only by 10 days post-fertilization (de Esch et al. 2012). This implies that in case compounds tested in the FET test are likely to induce severe neurodevelopmental effects, only part of the endpoints of neurotoxicity may be observable in 96-h-old embryos (Zindler et al. 2019a, b, 2020a, b). For some endpoints (such as swimming assays and anxiety tests), specific test setups might be required (Selderslaghs et al. 2009, 2012, 2013; Zindler et al. 2019a, 2020a, b).

## Conclusions

The present study aimed to differentiate between endpoints indicative of general or more specific pathologies. In any case, the present analysis of endpoints provides clear evidence that the fish embryo acute toxicity (FET) test can provide significantly more detailed information about the test compounds than originally planned for the OECD guideline 236. By the addition of an open list of further endpoints to the core observations specified by the original OECD guideline, the present communication was able to develop different endpoint profiles for the test compounds, even though the final “adverse outcome” of various pathways might ultimately be the same. Although a quite rudimentary type of “toxicity fingerprinting”, the syndrome originating from the collection of a full set of observations may well be of interest for regulatory purposes in terms of defining environmentally relevant threshold values. In combination with in-depth literature analysis, the present study also documents the usefulness of the FET for the development adverse outcome pathways (AOPs) for specific (classes of) test compounds. Based on the numerous advantages of the zebrafish as a test organism, and given its simplicity, versatility, reproducibility, and complementarity with other systems, the FET test has received increasing attention over many years and will continue to do so. However, future research would benefit greatly from the creation of a FET test endpoint database, allowing the comparison of effects, and from the use of a unified scoring system. An established, comprehensive nomenclature for different endpoints would make results obtained from different laboratories more comparable and allow for not only a more conclusive interpretation, but also a more in-depth understanding of observations.

## Supplementary information

ESM 1 (DOCX 71 kb)

## Data Availability

Original datasets of the current study and analyses generated are available in the BioStudies repository (https://wwwdev.ebi.ac.uk/biostudies/EU-ToxRisk/).
