# Supplementary material for: Specificity of time- and dose-dependent morphological endpoints in the fish embryo acute toxicity (FET) test for substances with diverse modes of action: the search for a “fingerprint”
Source: Environ Sci Pollut Res Int. 2021 Oct 13;29(11):16176–92. doi: 10.1007/s11356-021-16354-4 (PMC8827326; doi:10.1007/s11356-021-16354-4)
Supplement: Supplementary file 1 — (DOCX 71 kb) [file 11356_2021_16354_MOESM1_ESM.docx]

**Supplemental materials to:**

**Specificity of time- and dose-dependent morphological endpoints in the acute fish embryo toxicity test (FET) for substances with diverse modes-of-action: The search for a “fingerprint”**

Rebecca von Hellfeld*, Pauline Pannetier, Thomas Braunbeck*

University of Heidelberg, Center for Organismal Studies, Aquatic Ecology and Toxicology Section, Im Neuenheimer Feld 504, 69120 Heidelberg, Germany

**Contents:**

**Supplemental Materials Table 1.** Physicochemical properties of the compounds tested for their toxicity to embryos of the zebrafish (Danio rerio) in the FET according to OECD TG 236 (von Hellfeld et al. 2020)

**Supplemental Materials Table 2.** Application(s), affected organ(s) and biological effects of the compounds tested for their toxicity to embryos of the zebrafish (Danio rerio) in the FET according to OECD TG 236 (von Hellfeld et al. 2020)

**Supplemental Materials Table 1.** Physicochemical properties of the compounds tested for their toxicity to embryos of the zebrafish (*Danio rerio*) in the FET according to OECD TG 236 (von Hellfeld et al. 2020)

| **Compound** | **Water solubility** ^҂^ | **Log K_OW_ ^#^** | **Stability** | **Behavior in Water** |
| --- | --- | --- | --- | --- |
| Acrylamide | 390 mg/ml (25°C) | -0.67 | Hydrolysis: not likely (very slow). Biodegradation in water likely. River die-away test: 90 % reduction within 150 h ^#^ | N/A |
| Carbaryl | 110 mg/L (22 °C) | 2.36 | Biodegradation in sea water: 96 h. Half-life in water: 1.7 days. Hydrolysis: half-life at 20°C and pH 7 1.8 days ^#^. Hydrolyzed by various microorganisms. Not rapidly photodegraded * | Expected to adsorb to suspended solids and sediment ^#^ |
| Clofibrate | Insoluble | N/A | Not readily biodegradable ** | N/A |
| Colchicine | 45 g/L (25 °C) | 1.03 | Lacks functional groups for hydrolysis^#^. Readily biodegradable ** | Expected to adsorb to suspended solids and sediment ^#^ |
| Hexachlorophene | 140 mg/L (25 °C) | 7.54 | Lacks functional groups for hydrolysis. Photolysis: possible, absorbs 302 and 312 nm at 7 and 8 pH, respectively ^#^ | Moderate - high bioaccumulation. Expected to adsorb to suspended solids and sediment ^#^ |
| Ibuprofen | 21 mg/L (25 °C) | 3.97 | Hydrolysis: carboxylic acids generally resistant. Biodegradation in lakes: half-life 20 days. Mostly in anionic form at pH 5 - 9 – volatilization possible ^#^ | Expected to adsorb to suspended solids and sediment ^#^ |
| MPP+ | 10 mg/ml (25 °C) | N/A | Water-soluble iodide salt ^+^ | N/A |
| Paracetamol | 14 g/L (20 °C) | 0.46 | Lacks functional groups for hydrolysis ^#^. Readily biodegradable ** | Not expected to adsorb to suspended solids and sediment ^#^ |
| Paraquat | 700 mg/ml (20 °C) | -4.22 | Hydrolysis and direct photolysis not expected ^#^ | Expected to adsorb to suspended solids and sediment ^#^ |
| Polychlorinated Biphenyl 180 | Insoluble | N/A | Chemically very inert ^#^ | N/A |
| Rifampicin | 1400 mg/L (25 °C) | 4.24 | 10 mg/ml rifampicin: DMSO at 20°, pH 8: about 8 months ^+^. Not readily biodegradable ** | N/A |
| Rotenone | 0.17 mg/L (25 °C) | 4.10 | Biodegradation: conditional. First-order decay curve: half-life 10.3 d in cold water, 0.94 d in warm water. Photolysis: summer half-life 191 d at 2m depth, 21 h at surface. Lacks functional groups for hydrolysis ^#^ | (Very) high bioaccumulation. Expected to adsorb to suspended solids and sediment ^#^ |
| Sulfisoxazole | 300 mg/L (37 °C) | 1.01 | No hydrolysis of structurally similar compound. Direct photodegradation of anionic form ^#^ | Not expected to adsorb to suspended solids and sediments |
| Taxol | Insoluble | N/A | Not readily biodegradable ** | N/A |
| Tebuconazole | 36 mg/L (20 °C, pH 5-9) | 3.70 | Lacks functional groups for hydrolysis and experiments show stability in pure water ^#^ | Expected to adsorb to suspended solids and sediment ^#^ |
| Tolbutamide | 109 mg/L (37 °C) | 2.34 | Not readily biodegraded ** | N/A |
| Triphenylphosphate | 1.9 mg/L (25 °C) | 4.59 | Biodegradation half-life: 2 - 4 d. Volatilization from surface water expected: 20 - 152 d. Bioconcentration factor: 132 - 573 suggest high bioaccumulation ^#^ | Not expected to adsorb to suspended solids and particles |
| Valproic acid | 1.3 mg/L (22 °C) | 2.75 | No volatilization in anionic form, pH 5-9. Lacks functional groups for hydrolysis ^#^. Readily biodegradable ** | N/A |

N/A: No information available for the compound in question.

Data sources:

* Metabolic Pathways of Agrochemicals (Roberts and Hutson 1999): Roberts, T., & Hutson, D. (1999). Azoles and Analogues. In *Metabolic Pathways of Agrochemicals: Part 2: Insecticides and Fungicides* (pp. 1011–1104).
** Canadian Institute of Health research - DrugBank (version 5.1.1, released: 03.07.2018): <https://www.drugbank.ca/>
# US National Library of Medicine – ToxNet (now PubChem): <https://www.nlm.nih.gov/toxnet>
+ Sigma Aldrich MSDS specification sheets: <https://www.sigmaaldrich.com/germany.html>
҂ National Center for Biotechnology Information – PubChem: <https://pubchem.ncbi.nlm.nih.gov/>

**Supplemental Materials Table 2.** Application(s), affected organ(s) and biological effects of the compounds tested for their toxicity to embryos of the zebrafish (*Danio rerio*) in the FET according to OECD TG 236 (von Hellfeld et al. 2020)

| **Compound** | **Weight (g/Mol)** | **Chemical class** | **Application(s)** | **Affected organ(s)** | **Biological effects** |
| --- | --- | --- | --- | --- | --- |
| Acrylamide | 71.08 | Amide | Plastic thickener | Liver, nervous system | Endocrine disruption (Tyl and Friedman 2003)  Potassium channel inhibition (Faria et al. 2018) |
| Carbaryl | 201.22 | Carbamate | Insecticide | Nervous system | AChE inhibition (Schock et al. 2012)  CYP450 induction (Slaninova et al. 2009)  Competitive binding to melatonin receptors (Popovska-Gorevski et al. 2017) |
| Clofibrate | 242.70 | Fibrate | Antihyperlipidemic | Liver | PPARα agonist (Den Broeder et al. 2015)  CYP 450 induction (Laville et al. 2004) |
| Colchicine | 399.44 | Alkaloid | Anti-inflammatory, antineoplastic | Neuronal system | Microtubule inhibition *via* tubulin binding (Avila et al. 1987)  Neutrophil adhesion inhibition (Dalbeth et al. 2014) |
| Hexachlorophene | 406.89 | Organochlorine | Fungicide, acaricide,  antibiotic, flukicide | Liver, kidney, nervous system | Membrane-bound electron transport chain inhibition (Zheng et al. 2012)  Lesions in white matter (Tripier et al. 1981) |
| Ibuprofen | 206.285 | Proionic acid | Anti-inflammatory | Kidney, nervous system, intestine | Direct COX inhibition (David and Pancharatna 2009a)  PPARα and γ activation (Puhl et al. 2015) |
| Paracetamol | 151.165 | *p*-Aminophenol | Antipyretic | Liver, kidney, nervous system | Oxidative stress promotion (Du et al. 2016a)  Indirect COX-2 inhibition (David and Pancharatna 2009b) |
| Paraquat | 257.158 | Bipyridylium | Herbicide | Lung, liver, kidney, neuronal system | Oxidative stress promotion (Slaninova et al. 2009; Lushchak 2016)  Permanent neuronal damage (Parkinson’s) (Bretaud et al. 2004) |
| Polychlorinated biphenyl 180  (PCB 180) | 395.306 | Non-volatile chlorinated hydrocarbon | Industrial coolant, plasticizer (banned) | Liver | Glutamate-NO-cGMP pathway inhibition (Llansola et al. 2009) |
| Rifampicin | 822.953 | Ansamycin | Antibiotic | Liver, kidney | RNA polymerase inhibition (Campbell et al. 2001)  CYP450 induction (Mahatthanatrakul et al. 2007) |
| Rotenone | 394.423 | Furanocoumarin | Insecticide, acaricide, piscicide | Liver, kidney, nervous system | Oxidative stress promotion (Slaninova et al. 2009; Pinho et al. 2013; Wang et al. 2017)  Microtubule formation disruption (Cheng and Farrell 2007) |
| Sulfisoxazole | 267.303 | Sulfonamide | Antibiotic | Bacterial | Bacterial folic acid synthesis inhibition (Hong et al. 1995)  CYP450 inhibition (Hong et al. 1995) |
| Taxol (Paclitaxel) | 853.918 | Taxane | Chemotherapy drug | Neuronal system | Stabilization of microtubules (tubulin β-1 chain inhibition) (Brito et al. 2008)  Oxidative stress promotion (Lisse et al. 2016) |
| Tebuconazole | 307.822 | Triazole | Fungicide | Liver | Cell wall production disruption (Sancho et al. 2010; Liu et al. 2013)  CYP450 inhibition (Yang et al. 2018)  Oxidative stress promotion (Yang et al. 2018)  AChE inhibition (Altenhofen et al. 2017)  Endocrine disruption (Sancho et al. 2010) |
| Tolbutamide | 270.347 | Sulfonyl urea | Hypoglycemic drug | Liver, kidney | Potassium channel closure of ß-cells (Zhou et al. 2009) |
| Triphenylphosphate | 326.288 | Phenol | Flame retardant, plasticizer | Liver, neuronal system | Endocrine disruption (Liu et al. 2016)  Metabolic disruption (Du et al. 2016b)  PPAR γ activation (Du et al. 2016b)  CYP450 inhibition (Isales et al. 2015) |
| Valproic acid | 144.211 | Fatty acid | Anti-epileptic drug | Liver, nervous system | HDAC inhibition (Chateauvieux et al. 2010; Godhe-Puranik et al. 2013)  Sodium channel protein inhibition (Chateauvieux et al. 2010; Godhe-Puranik et al. 2013)  GABA transaminase inhibition (Chateauvieux et al. 2010; Godhe-Puranik et al. 2013) |

**References**:

Altenhofen S, Nabinger DD, Wiprich MT, et al (2017) Tebuconazole alters morphological, behavioral and neurochemical parameters in larvae and adult zebrafish (*Danio rerio*). Chemosphere 180:483–490. https://doi.org/10.1016/j.chemosphere.2017.04.029

Avila J, Serrano L, Maccioni RB (1987) Regulatory aspects of the colchicine interactions with tubulin. Mol Cell Biochem 73:29–36. https://doi.org/10.1007/BF00229373

Bretaud S, Lee S, Guo S (2004) Sensitivity of zebrafish to environmental toxins implicated in Parkinson’s disease. Neurotoxicol Teratol 26:857–864. https://doi.org/10.1016/j.ntt.2004.06.014

Brito DA, Yang Z, Rieder CL (2008) Microtubules do not promote mitotic slippage when the spindle assembly checkpoint cannot be satisfied. J Cell Biol 182:623–629. https://doi.org/10.1083/jcb.200805072

Campbell EA, Korzheva N, Mustaev A, et al (2001) Structural mechanism for rifampicin inhibition of bacterial RNA polymerase. Cell 104:901–912. https://doi.org/10.1016/S0092-8674(01)00286-0

Chateauvieux S, Morceau F, Dicato M, Diederich M (2010) Molecular and therapeutic potential and toxicity of valproic acid. J Biomed Biotechnol 2010:1–18. https://doi.org/10.1155/2010/479364

Cheng WW, Farrell AP (2007) Acute and sublethal toxicities of rotenone in juvenile rainbow trout (Oncorhynchus mykiss): Swimming performance and oxygen consumption. Arch Environ Contam Toxicol 52:388–396. https://doi.org/10.1007/s00244-006-0051-1

Dalbeth N, Lauterio TJ, Wolfe HR (2014) Mechanism of action of colchicine in the treatment of gout. Clin Ther 36:1465–1479. https://doi.org/10.1016/j.clinthera.2014.07.017

David A, Pancharatna K (2009a) Developmental anomalies induced by a non-selective COX inhibitor (ibuprofen) in zebrafish (*Danio rerio*). Environ Toxicol Pharmacol 27:390–395. https://doi.org/10.1016/j.etap.2009.01.002

David A, Pancharatna K (2009b) Effects of acetaminophen (paracetamol) in the embryonic development of zebrafish, *Danio rerio*. J Appl Toxicol 29:597–602. https://doi.org/10.1002/jat.1446

Den Broeder MJ, Kopylova VA, Kamminga LM, Legler J (2015) Zebrafish as a model to study the role of peroxisome proliferating-activated receptors in adipogenesis and obesity. PPAR Res 2015:1–11. https://doi.org/10.1155/2015/358029

Du K, Ramachandran A, Jaeschke H (2016a) Oxidative stress during acetaminophen hepatotoxicity: Sources, pathophysiological role and therapeutic potential. Redox Biol 10:148–156. https://doi.org/10.1016/j.redox.2016.10.001

Du Z, Zhang Y, Wang G, et al (2016b) TPhP exposure disturbs carbohydrate metabolism, lipid metabolism, and the DNA damage repair system in zebrafish liver. Sci Rep 6:1–10. https://doi.org/10.1038/srep21827

Faria M, Ziv T, Gómez-Canela C, et al (2018) Acrylamide acute neurotoxicity in adult zebrafish. Sci Rep 8:1–14. https://doi.org/10.1038/s41598-018-26343-2

Godhe-Puranik Y, Thorn CF, Lambra JK, Leeder JS (2013) Valproic acid pathway: pharmacokinetics and pharmacodynamics. Pharmacogenet Genomics 23:236–241. https://doi.org/10.1097/FPC.0b013e32835ea0b2.Valproic

Hong YL, Hossler PA, Calhoun DH, Meshnick SR (1995) Inhibition of recombinant Pneumocystis carinii dihydropteroate synthetase by sulfa drugs. Antimicrob Agents Chemother 39:1756–1763. https://doi.org/10.1128/AAC.39.8.1756

Isales GM, Hipszer RA, Raftery TD, et al (2015) Triphenyl phosphate-induced developmental toxicity in zebrafish: Potential role of the retinoic acid receptor. Aquat Toxicol 161:221–230. https://doi.org/10.1016/j.aquatox.2015.02.009

Laville N, Aït-Ässa S, Gomez E, et al (2004) Effects of human pharmaceuticals on cytotoxicity, EROD activity and ROS production in fish hepatocytes. Toxicology 196:41–55. https://doi.org/10.1016/j.tox.2003.11.002

Lisse TS, Middleton LJ, Pellegrini AD, et al (2016) Paclitaxel-induced epithelial damage and ectopic MMP-13 expression promotes neurotoxicity in zebrafish. Proc Natl Acad Sci 113:2189–2198. https://doi.org/10.1073/pnas.1525096113

Liu C, Wang Q, Liang K, et al (2013) Effects of tris(1,3-dichloro-2-propyl) phosphate and triphenyl phosphate on receptor-associated mRNA expression in zebrafish embryos/larvae. Aquat Toxicol 128–129:147–157. https://doi.org/10.1016/j.aquatox.2012.12.010

Liu X, Jung D, Jo A, et al (2016) Long-term exposure to triphenylphosphate alters hormone balance and HPG, HPI, and HPT gene expression in zebrafish (*Danio rerio*). Environ Toxicol Chem 35:2288–2296. https://doi.org/10.1002/etc.3395

Llansola M, Piedrafita ÁB, Rodrigo ÁR, et al (2009) Polychlorinated biphenyls PCB 153 and PCB 126 impair the glutamate- nitric oxide-CgmpGlutamate – nitric oxide – cGMP pathway in cerebellar neurons in culture by different mechanisms. Chem Res Toxicol 23:813–820. https://doi.org/10.1007/s12640-009-9055-8

Lushchak VI (2016) Contaminant-induced oxidative stress in fish: a mechanistic approach. Fish Physiol Biochem 42:711–747. https://doi.org/10.1007/s10695-015-0171-5

Mahatthanatrakul W, Nontaput T, Ridtitid W, et al (2007) Rifampin, a cytochrome P450 3A inducer, decreases plasma concentrations of antipsychotic risperidone in healthy volunteers. J Clin Pharm Ther 32:161–167. https://doi.org/10.1111/j.1365-2710.2007.00811.x

Pinho B, Santos M, Fonseca-Silva A, et al (2013) How mitochondrial dysfunction affects zebrafish development and cardiovascular function: An in vivo model for testing mitochondria-targeted drugs. Br J Pharmacol 169:1072–1090. https://doi.org/10.1111/bph.12186

Popovska-Gorevski M, Dubocovich ML, Rajnarayanan R V. (2017) Carbamate insecticides target human melatonin receptors. Chem Res Toxicol 30:574–582. https://doi.org/10.1021/acs.chemrestox.6b00301

Puhl AC, Milton FA, Cvoro A, et al (2015) Mechanisms of Peroxisome Proliferator Activated Receptor γ Regulation by Non-steroidal Anti-inflammatory Drugs. Nucl Recept Signal 13:nrs.13004. https://doi.org/10.1621/nrs.13004

Roberts T, Hutson D (1999) Azoles and Analogues. In: Metabolic Pathways of Agrochemicals: Part 2: Insecticides and Fungicides. pp 1011–1104

Sancho E, Villarroel MJ, Fernández C, et al (2010) Short-term exposure to sublethal tebuconazole induces physiological impairment in male zebrafish (*Danio rerio*). Ecotoxicol Environ Saf 73:370–376. https://doi.org/10.1016/j.ecoenv.2009.09.020

Schock EN, Ford WC, Midgley KJ, et al (2012) The effects of carbaryl on the development of zebrafish (Danio rerio) embryos. Zebrafish 9:169–178. https://doi.org/10.1089/zeb.2012.0747

Slaninova A, Smutna M, Modra H, Svobodova Z (2009) A review: oxidative stress in fish induced by pesticides. Neuro Endocrinol Lett 30 Suppl 1:2–12. https://doi.org/NEL300709R01 [pii]

Tripier MF, Bérard M, Toga M, et al (1981) Hexachlorophene and the central nervous system - Toxic effects in mice and baboons. Acta Neuropathol 53:65–74. https://doi.org/10.1007/BF00697186

Tyl RW, Friedman MA (2003) Effects of acrylamide on rodent reproductive performance. Reprod Toxicol 17:1–13. https://doi.org/10.1016/S0890-6238(02)00078-3

von Hellfeld R, Brotzmann K, Baumann L, et al (2020) Adverse effects in the Fish Embryo Acute Toxicity (FET) test - a catalogue of unspecific morphological changes *versus* more specific effects in zebrafish (*Danio rerio*) embryos. Environ Sci Eur 32:122. https://doi.org/10.1186/s12302-020-00398-3

Wang Y, Liu W, Yang J, et al (2017) Parkinson’s disease-like motor and non-motor symptoms in rotenone-treated zebrafish. Neurotoxicology 58:103–109. https://doi.org/10.1016/j.neuro.2016.11.006

Yang J Di, Liu SH, Liao MH, et al (2018) Effects of tebuconazole on cytochrome P450 enzymes, oxidative stress, and endocrine disruption in male rats. Environ Toxicol 33:899–907. https://doi.org/10.1002/tox.22575

Zheng Y, Zhu X, Zhou P, et al (2012) Hexachlorophene is a potent KCNQ1/KCNE1 potassium channel activator which rescues LQTs mutants. PLoS One 7:1–9. https://doi.org/10.1371/journal.pone.0051820

Zhou S-F, Zhou Z-W, Yang L-P, Cai J-P (2009) Substrates, inducers, inhibitors and structure-activity relationships of human Cytochrome P450 2C9 and implications in drug development. Curr Med Chem 16:3480–675. https://doi.org/CMC - AbsEpub - 001 [pii]
